# Serious adverse effect of low-dose dexmedetomidine under spinal anesthesia: A case report and literature review

**DOI:** 10.5339/qmj.2025.117

**Published:** 2025-12-14

**Authors:** Muhammad Firas Alhammad, Muhammad Jaffar Khan, Arunabha Karmakar, Hazim Kassas, Yasser Hammad, Yasir Ahmed, Mohamad Talal Basrak, Tarek Tageldin, Adnan Alshami, Nabil Shallik

**Affiliations:** 1 Anaesthesia, ICU and Perioperative Medicine Department, Hamad Medical Corporation, Doha, Qatar; 2 Tower Health, West Reading, Pennsylvania, United States of America; 3 Department of Anesthesia, Intensive and Emergency Medicine, and Pain Management, Helios Klinikum Cuxhaven, Lower Saxony, Germany; 4 Clinical Anaesthesiology Department, Weill Cornell Medical College in Qatar, Doha, Qatar; 5 Clinical Anaesthesiology Department, College of Medicine, Qatar University, Doha, Qatar; 6 Clinical Anaesthesia and SICU Department, Tanta University, Tanta, Egypt *Email: malhammad1@hamad.qa

**Keywords:** Dexmedetomidine, bradycardia, pericardiac arrest, spinal anesthesia, neuraxial anesthesia

## Abstract

**Background:**

Dexmedetomidine is a highly selective α_2-_adrenergic agonist with sedo-analgesic properties. It has been approved by the United States Food and Drug Administration (FDA) for sedation of critically ill patients, facilitation of awake procedures, and management of patient agitation. However, its use has been occasionally associated with adverse effects like bradycardia, hypotension, and asystole. The risk of these effects, particularly cardiac arrest, is increased in the elderly, patients with multiple comorbidities, and administration of higher doses of the drug (especially loading doses). However, perioperative pericardiac arrest with only low-dose dexmedetomidine infusion, in a young healthy male, has not been previously reported.

**Case Presentation:** We report a 33-year-old male, ASA 1 E, who underwent open reduction and internal fixation of a left ankle fracture under spinal anesthesia. He was sedated intraoperatively with intravenous dexmedetomidine, infused at 0.2 μg/kg/h, without a loading dose. Approximately 90 minutes into the procedure, he had severe bradycardia and hypotension, leading to a peri-arrest situation. He was successfully revived with intravenous atropine and ephedrine and completed the surgery uneventfully.

**Conclusion:**

We opine that the use of dexmedetomidine as a sedative agent in patients under spinal anesthesia requires heightened caution due to the risk of major adverse cardiac events. Our case exemplifies that this risk persists even in healthy young patients, at low drug infusion rates, and even after avoiding a loading dose.

## 1. INTRODUCTION

Approximately 100 million non-cardiac surgeries are performed globally each year, among which 0.5% to 1.0 % of patients experience perioperative cardiac complications, including non-fatal cardiac arrest, non-fatal myocardial infarction, or cardiac death.^1^ The 7th National Audit Project from the United Kingdom (NAP 7) reported that bradyarrhythmias accounted for approximately 9.4% of cases of perioperative cardiac arrests.^2^ However, bradycardic cardiac arrest also had the highest rate of successful resuscitation and survival to hospital discharge (86% and 60%, respectively).^3^ One study noted the incidence of intraoperative bradycardia as 42% when defined as an intraoperative heart rate more than 30% lower than the patient’s mean nighttime heart rate, and 43% when defined as an intraoperative heart rate below 45 beats per minute.^4^ Registration documents from the European Medicines Agency and the U.S. Food and Drug Administration have reported a heightened incidence of bradycardia in patients aged over 65 years.^5^ Many pharmacological agents used in the perioperative period may contribute to this either as a dose-dependent side effect or a toxic effect. Dexmedetomidine hydrochloride is a sedative and analgesic agent, indicated for sedation in non-intubated patients, and for sedation before or during surgical or medical procedures. It has a potent, highly selective, centrally acting, α_2_ adrenergic agonistic effect (α_1_: α_2_ = 1:1620).^6^ Additional benefits include a decrease in postoperative shivering, nausea, and vomiting.^7,8^ One remarkable property of dexmedetomidine is its minimal potential to cause respiratory depression compared to other sedatives (Table 1).^6^

For neuraxial anesthesia, intravenous dexmedetomidine has also been found to prolong the duration of sensory blockade and improve the quality of post-operative analgesia.^9,10^

However, dexmedetomidine can have notable hemodynamic side effects such as bradycardia and, in rare instances, even cardiac arrest, particularly in elderly patients. Hemodynamic instability induced by dexmedetomidine typically presents as hypotension and bradycardia and occurs in a dose-dependent manner.^11^

We report to our knowledge, the first instance of bradyarrhythmia leading to pericardiac arrest in a healthy young patient under spinal anesthesia, after receiving the lowest recommended dose of dexmedetomidine infusion for sedation, without any initial loading dose. Our case serves to remind us that while intravenous infusion of dexmedetomidine can achieve effective sedation during spinal anesthesia, it is important to note the potential for hemodynamic instability, including occurrences of bradycardia, hypotension, and transient hypertension.^12^ The likelihood of experiencing intraoperative bradycardia or cardiac arrest rises in the presence of concurrent comorbidities.^13^ Cardiovascular disease and the utilization of antihypertensive drugs may amplify the impact of vagal bradycardia. Furthermore, medications designed to reduce heart rate, such as beta-blockers, could heighten the likelihood of intraoperative bradyarrhythmia progressing to cardiac arrest.^14^ In our case report, it’s noteworthy that the patient had no underlying medical conditions, nor was he of advanced age. Additionally, the intraoperative sedation with dexmedetomidine was performed with the lowest recommended maintenance dose^15^ and without a loading dose.

## 2. CASE PRESENTATION

A 32-year-old male presented to the emergency department reporting pain and swelling in his left ankle after a twisting injury sustained while playing football. He had no significant medical history or ongoing treatments. Physical examination revealed moderate swelling and deformity in the left ankle, with soft compartments and no distal neurovascular deficits. Radiograph confirmed a bimalleolar fracture of the left ankle. In the emergency setting, a below-knee back-slab was applied, and the case was subsequently reviewed by the orthopedic surgical team. Diagnosis of a left ankle bimalleolar fracture was established, and the decision was made to proceed with open reduction and internal fixation.

Preoperative assessment revealed the patient to be in good health, with no notable abnormalities on physical examination. The patient, classified as ASA 1E, weighed 70 kg and was 170 cm tall. Laboratory tests yielded results within normal ranges. Informed consent was obtained for spinal anesthesia, with general anesthesia planned as a contingency.

On the day of surgery, standard ASA monitoring was initiated, and intravenous access was verified. The patient’s baseline vital signs are demonstrated in Table 2. Spinal anesthesia was performed in the sitting position using a 25-gauge spinal needle and a mixture of 3 mL hyperbaric bupivacaine 0.5% with fentanyl 15 μg. Co-loading with crystalloid (Ringer’s lactate) infusion was used during spinal anesthesia.

Following successful spinal anesthesia, the patient was repositioned supine and remained hemodynamically stable for the remainder of the procedure. After injection, the depth and height of the subarachnoid block were assessed with the patient reporting loss of motor power and loss of sensation to cold temperature (ethyl chloride spray). The upper dermatomal level of block was noted to be T8. A simple face mask provided 5 L/min of oxygen. We had drawn 200 μg of dexmedetomidine (2 mL Precedex™) from a vial and diluted it in 48 mL of 0.9% saline to achieve a concentration of 4 μg/mL, and it was started after block onset as an intravenous infusion at 0.2 μg/kg/h.

Surgery was performed after applying a tourniquet to the left thigh at a pressure of 250 mm Hg. A 6 cm incision over the lateral malleolus, followed by dissection to expose the fracture site and achieve anatomic reduction with clamps and plate fixation, was carried out. A similar procedure was performed on the medial malleolus, utilizing wire fixation. Sedation was maintained via a dexmedetomidine infusion at 0.2 μg/kg/h without a loading dose. Bispectral Index (BIS) monitoring was utilized for assessing the depth of sedation.

Approximately 90 minutes into the procedure, with the tourniquet inflated, the patient experienced sudden bradycardia, with a heart rate of 20 beats/min and a reduced conscious level. Fortunately, the patient’s airway remained patent as evidenced by visible chest expansion, normal capnography, and normal SpO_2_ of 97%. ECG showed sinus bradycardia, prompting administration of intravenous atropine (0.5 mg) in addition to cessation of dexmedetomidine infusion. Surgical manipulation was halted, and a second dose of atropine (0.5 mg) was administered with ephedrine (9 mg). The patient’s heart rate and blood pressure improved to 70 beats/min and 107/55 mm Hg, respectively. A rapid infusion of plasma protein fraction 5% 250 mL was delivered intravenously for intravascular volume expansion. Oxygen flow rate was increased up to 15 L/min, using a simple face mask. The improvement in the patient’s clinical status (Table 2) and level of consciousness did not necessitate epinephrine usage. Following stabilization, surgery resumed and was completed without further incident. The tourniquet was released with a total inflation time of 81 minutes, the surgical duration lasted 120 minutes, and the patient was transferred to the postoperative care unit before being discharged to the ward.

## 3. DISCUSSION

To our knowledge, this is the first reported instance of pericardiac arrest bradyarrhythmia in a healthy young male during an open reduction and internal fixation of a bimalleolar ankle fracture under spinal anesthesia while receiving the lowest recommended dose of dexmedetomidine infusion for sedation, without an initial loading dose. Towards the middle of the surgery, he had developed sudden bradycardia and hypotension, which were immediately reversed with atropine and ephedrine.

The use of dexmedetomidine has significantly expanded in the perioperative setting with evolving evidence. For the initiation of procedural sedation with dexmedetomidine, the U.S. Food and Drug Administration (FDA) recommends the following protocols.^15^

- For invasive procedures or awake fiberoptic intubation, a recommended loading infusion dosage of 1 μg/kg over 10 minutes is advised.
- For invasive procedures such as ophthalmic surgery, a recommended loading infusion dosage of 0.5 μg/kg over 10 minutes is recommended.
- For all procedures except awake fiberoptic intubation, it is generally recommended to initiate a maintenance infusion dosage of 0.6 μg/kg/h and titrate as necessary to achieve the desired clinical effect, with dosages ranging from 0.2 to 1 μg/kg/h.
- For awake fiberoptic intubation, a maintenance infusion dose of 0.7 μg/kg/h is recommended until the endotracheal tube is secured.

Bradycardia is a frequent adverse reaction associated with dexmedetomidine, with an incidence ranging from 10% to 30%, depending on the dose administered.^16,17^ The reported incidence of bradycardia following spinal anesthesia coupled with intravenous dexmedetomidine administration ranges from 20% to 30%.^18,19^ In human studies employing intravenous boluses of dexmedetomidine, administration of small boluses (0.25–1 μg/kg) has resulted in reductions in blood pressure (BP) and cardiac output (CO). Conversely, larger boluses (1–4 μg/kg) have elicited a transient elevation in BP along with occasional profound reflex bradycardia.^11^ The impact of dexmedetomidine on heart rate was most accurately depicted by a non-linear model.^20^ The correlation between cardiac arrest and low concentrations of dexmedetomidine lacks a clear explanation. The risk of cardiac arrest is not inherently linked to the blood concentration of dexmedetomidine. Additionally, the definition of intraoperative bradycardia in patients under general anesthesia lacks consensus. Different criteria are employed in various studies, resulting in incomparable outcomes. The precise criteria for defining physiologically significant intraoperative bradycardia also remain undetermined. Bradycardia in the general adult population is commonly defined as a heart rate below 60 beats per minute.^21^

Heart rate, in conjunction with stroke volume, plays a significant role in determining cardiac output. Bradycardia can lead to a reduction in cardiac output and subsequently diminish oxygen delivery to vital organs.^22^ Intraoperative bradycardia can stem from various causes, including reflexes such as the Oculo-cardiac reflex, Bezold-Jarisch reflex, and baroreceptor reflex. If left unmanaged, acute bradycardia can swiftly progress to asystole, leading to hemodynamic collapse. Common triggers of bradycardia include hypoxia and medication administration during anesthesia, such as beta-blockers, calcium channel blockers, opioids, and alpha 2 agonists. Additionally, acute vagal stimulation and neuraxial anesthesia can also precipitate bradycardia. Furthermore, acute cardiac events, such as myocardial infarction affecting the blood supply to the SA node or a high-degree AV node block, may be causative.

When managing intraoperative bradycardia, simultaneous interventions are necessary. These include determining the hemodynamic significance of the event and scanning the surgical field to identify any red flags (uncontrolled bleeding, for example), and promptly notifying the surgeon as needed. Additionally, it’s crucial to ensure that the patient’s ventilation and oxygenation are maintained. Finally, pharmacological treatment or transcutaneous pacing should be provided as required. In all circumstances, the treatment of symptomatic or hemodynamically significant bradycardia should be initiated without delay as per guidelines.^21^

Prophylactic anticholinergics may help prevent bradycardia in spinal anesthesia with dexmedetomidine infusion.^16,18^ While atropine remains an option for treating bradycardia, its effectiveness may be inadequate at certain dosages.^23^ A potential reduction in atropine’s positive chronotropic effects could occur when dexmedetomidine or propofol is utilized alongside spinal anesthesia. Consequently, caution is advised when considering atropine premedication.^16^ Clonidine, an α-adrenergic receptor agonist with high selectivity for α_2_, has also been documented to reduce the heart rate response to intravenous atropine. Dexmedetomidine boasts approximately eight times greater α_2_ selectivity compared to clonidine. This heightened selectivity results in a more potent sympathetic blockade, thereby leading to a stronger attenuation of atropine’s effects.^24^ Additionally, at the time of the event, there was a discrepancy between the heart rate of 20 bpm and the blood pressure reading of 70/42 mm Hg, as the blood pressure measurement did not coincide with the moment of acute bradycardia. While heart rate was continuously monitored via ECG, blood pressure was assessed intermittently using a non-invasive cuff at regular intervals.

Our patient’s baseline physically active status may have also predisposed him to bradycardia due to high vagal tone, even when his pre-operative baseline heart rate was within normal range.

Research indicates that loading doses of 0.5 to 1.0 μg/kg dexmedetomidine over 10 minutes, followed by an infusion at a rate of 0.2 to 0.7 μg/kg/h, offer effective sedation and are well-tolerated by elderly patients.^25^ Hemodynamic effects of dexmedetomidine following short-term (2, 5, or 10-minute) infusions have been documented and outlined at doses ranging from 0.25 to 4 μg/kg.^17^ Previous studies have demonstrated that peak decrease in heart rate occurs at 3 minutes and persists for 11 minutes following the infusion of 0.25 to 2.0 μg/kg of dexmedetomidine over 2 minutes in healthy adult volunteers.^11^ Bradycardia may serve as a limiting factor in administering dexmedetomidine at higher concentrations, particularly in patients with pre-existing bradycardia or those who benefit from higher heart rates, such as individuals with dilated cardiac failure. Cardiac depression may ensue when the plasma concentration of dexmedetomidine surpasses 1.2 ng/mL.^26^ The median effective dose (ED 50) of dexmedetomidine resulting in bradycardia in a recent cohort study published by Yang et al. was 1.97 μg/kg/h, which was higher than the FDA-recommended dose.^27^ Research has revealed a narrow hysteresis between plasma concentration and heart rate effects, indicating a rapid heart rate response to changes in plasma concentration.^17^ Although dexmedetomidine has a significant impact on hemodynamic parameters in elderly patients, the pharmacokinetic profile of dexmedetomidine remains unaltered by age.^6^ A case documented dexmedetomidine-associated bradycardia, which had advanced to pulseless electrical activity (PEA) in a 74-year-old male. He had developed post-operative myocardial infarction 3 days after repair of an abdominal aortic aneurysm and required sedation for agitation. Dexmedetomidine started at 0.11 μg/kg/h, and the administered dose was gradually up-titrated to reach 0.7 μg/kg/h over 6 hours. Despite stable initial vital signs, he had progressed to severe bradycardia and PEA. Cessation of dexmedetomidine with administration of atropine had revived the patient.^28^ Dexmedetomidine-related cardiac arrest had also been reported in a 76-year-old woman despite having a permanent pacemaker. She had been scheduled for surgery to remove her infected permanent pacemaker due to staphylococcal bacteremia. An infusion of dexmedetomidine (1 μg/kg) was initiated as a loading dose for 20 minutes, which would translate to a rate of 3 μg/kg/h. Approximately 15 minutes after the start of infusion, she began coughing, developed dyspnea, and rapidly lost consciousness. Unfortunately, she was unable to be revived.^29^ Similarly, cardiac arrest was reported in the critical care unit during dexmedetomidine-based sedation of a 64-year-old woman with multiple comorbidities (hypertension, diabetes mellitus, and asymptomatic first-degree AV block) post low anterior resection of the rectum. Despite not receiving any loading dose, she progressed to second degree and ultimately complete heart block. She was successfully reverted to sinus rhythm with cardiac massage.^30^ In another report, a 56-year-old male patient received dexmedetomidine at a rate of 0.3 μg/kg/h, which was lower than the recommended dose, for sedation in an intensive care unit. He had previously undergone open cardiac surgery, and atrial pacing had been consistently maintained at a fixed rate of 90 beats/min.^31^ After dexmedetomidine infusion, he developed prolongation of the PQ interval followed by complete atrioventricular block and cardiac arrest. He was revived after 15 minutes of cardiopulmonary resuscitation and later discharged without any neurological complications.^31^

Cephalic extension of spinal blockades (high spinal) can potentially block the sympathetic outflow from the upper thoracic spinal cord and cause hypotension and bradycardia. Additionally, it may also cause a transient loss of consciousness. However, it is less likely to have occurred in our patient as there was no associated extension of sensory or motor block. In addition, the rapid correction of bradycardia in response to atropine suggests a different etiology than a high spinal.

Some factors that add to the risk of bradycardia associated with dexmedetomidine during spinal anesthesia are baseline heart rate (HR) and tourniquet time.^32^ Dexmedetomidine may mitigate the hyperdynamic response in patients undergoing lower extremity surgery with tourniquet application. Consequently, the observed heightened risk of bradycardia development with prolonged tourniquet time could be attributed to the effects of dexmedetomidine.^33^ Also, severe tourniquet pain triggering a vasovagal response could be considered as a potential explanation for the event. However, in our case, this scenario was deemed unlikely. The level of the block was assessed promptly after the patient stabilized, revealing a T8 level. For patients exhibiting initial bradycardia or undergoing surgeries anticipated to involve prolonged tourniquet application, anesthesiologists can mitigate the risk of bradycardia by reducing the loading dose of dexmedetomidine. Moreover, administering anticholinergic premedication has proven effective in preventing bradycardia.^34^

## 4. CONCLUSION

We believe that the use of dexmedetomidine as a sedative agent in patients undergoing spinal anesthesia requires heightened caution, as demonstrated in our case. Bradycardia with dexmedetomidine use is common, and significant hemodynamic instability is common in elderly patients, those with multiple comorbidities, high infusion dose, and use of a loading dose. However, the risk of peri-cardiac arrest persists even in healthy young patients, at low infusion rates, and even after avoiding the loading dose. Premedication with an anticholinergic like atropine may prevent serious bradycardia associated with intraoperative dexmedetomidine. However, it may on occasion not respond adequately and may necessitate the use of epinephrine.

## DATA AVAILABILITY

Data will be made available on request.

## ETHICS APPROVAL

This case report was submitted for review and approval by the Medical Research Centre of Hamad Medical Corporation and was approved with the ID MRC-04-25-056. The patient has provided written informed consent for the publication of this case report.

## ACKNOWLEDGEMENTS

All the authors have contributed to this manuscript and agree with its contents. Written informed consent was obtained from the patient before publishing this case report. The publication of this report has been approved by our institutional IRB (protocol ID MRC 04-25-056).

## CONFLICT OF INTEREST

The authors declare no conflicts of interest.

## Figures and Tables

**Table 1.** Commonly used sedatives in the perioperative period.

**Sl. No.**	**Agent**	**Pro**	**Con**
1	Propofol infusion	Good hypnotic, anti-seizure, and anti-emetic effects, and easily titratable to maintain depth of anesthesia.	No analgesic effect, painful infusion, may cause apnea at higher doses.
2	Midazolam	Very good sedative, anxiolytic, amnestic, relaxant, and anti-seizure effects.	Not easily titratable, respiratory depression.
3	Fentanyl	Very good sedative and analgesic effects.	Respiratory depression, pruritus, nausea, and vomiting can occur.

**Table 2.** Patients’ vital signs (heart rate [HR] in beats/min; blood pressure [BP]) in mm Hg before, during, and after treatment of the arrhythmia.

Baseline	HR	78
	BP	106/63
During arrhythmia	HR	20
	BP	70/42
After the correction of the arrhythmia	HR	70
	BP	107/55
